# What triggers seismicity thousands of kilometers away from a mainshock?

**DOI:** 10.1126/sciadv.aec4754

**Published:** 2026-06-24

**Authors:** Chao Huang, Jun Yang

**Affiliations:** Department of Civil Engineering, The University of Hong Kong, Hong Kong, China.

## Abstract

Remotely triggered earthquakes have emerged as a compelling yet puzzling phenomenon for advancing seismic-hazard forecasting. Here, we propose a unifying mechanism to explain their enigmatic behaviors, that is, the pore-pressure dynamics in fault gouge. Using a bounding surface model rooted in critical-state soil mechanics, we embed gouge deformation into a computational framework that tracks stress evolution under the perturbation of teleseismic waves. Our simulations reveal that dynamic loading drives pore-pressure increases several times larger than the applied stress amplitude, thus markedly weakening the fault and enabling failure under minimal perturbations. Also, we show that compressional components of the waves amplify pore pressure far more than shear components, explaining the superior triggering capacity of Rayleigh waves over Love waves. Given that pore pressure dissipates slowly in low-permeability gouge, weakening persists well beyond the transient forcing, promoting elevated seismicity after wave passage. Together, these results establish fault-gouge pore-pressure dynamics as a fundamental mechanism for remote earthquake triggering.

## Teaser

Pore-pressure surges in fault gouge under seismic waves reshape the understanding of remotely triggered earthquakes.

## INTRODUCTION

In recent decades, seismologists have documented a notable phenomenon: Large earthquakes can trigger seismicity far beyond conventional aftershock zones, sometimes thousands of kilometers from the mainshock (*1*). This phenomenon, known as remote earthquake triggering, is not sporadic but frequently observed across diverse tectonic settings (*2*–*5*). While most triggered events are small [*M* (magnitude) < 5] and pose little hazard, they offer rare opportunities to probe crustal stress states, with implications for earthquake forecasting and hazard assessment (*6*–*8*). Unlocking this potential requires understanding the physical mechanisms underlying remote triggering—a challenge that remains unresolved in earthquake science.

Pinpointing the mechanism of remote triggering hinges on a central question: What transmits failure stresses from a mainshock to seismicity thousands of kilometers away? Evidence points to seismic waves from the mainshock as the primary agent (*1*, *9*–*12*), supported by strong space-time correlations between teleseismic wave arrival and the onset of triggered events. This leads to the next question: Which wave properties control the triggering process?

Statistical analyses show that remote seismicity systematically increases with dynamic stress amplitude (*6*, *13*). Yet, locally, this link can break down. For example, the 1994 *M* 6.7 Northridge earthquake produced stronger peak ground motions at Long Valley Caldera than the 2002 *M* 7.9 Denali event, but only Denali triggered seismicity there (*14*). A subsequent study confirms no consistent tie between triggering rates and local peak ground velocity (*15*).

Wave frequency also critically influences triggering—but remains contentious. Rate-and-state friction theory predicts that high-frequency stresses (periods < fault nucleation times) should be less effective, owing to limited time for frictional state evolution, a prediction supported by laboratory experiments (*16*, *17*). Brodsky and Prejean (*18*) argued that low-frequency waves, at comparable amplitudes, penetrate deeper and thus more effectively promote failure—as seen in Denali’s Long Valley activity. Conversely, other field observations and laboratory work report stronger triggering by short-period, high-frequency pulses (*19*).

The type of surface wave further modulates triggering efficiency. Coulomb stress calculations indicate that Love waves impose larger stress changes than Rayleigh waves on vertical strike-slip faults through horizontal shear (*20*). Given the global prevalence of near-critical strike-slip faults (*21*), Love wave dominance might be expected. Yet, in many cases, triggered seismicity initiates during the later-arriving Rayleigh waves rather than the preceding Love waves. These contradictions expose a core gap: We still lack a clear mechanism explaining which teleseismic wave traits control the nucleation of remotely triggered earthquakes.

The hypothesis of triggering from seismic waves faces greater complexity with delayed events—earthquakes starting minutes to months after triggering waves pass (*22*–*24*). This phenomenon raises a central puzzle: How transient seismic wave stress produces delayed or prolonged fault failure?

Existing physical models attribute the delay to secondary processes where dynamic stresses alter fault properties. The rate-state friction framework, for example, incorporates a time-dependent decrease in the friction coefficient (*25*, *26*). However, this model struggles to explain delayed triggering resulting from high-frequency excitation, given the limited nucleation timescale (*16*). Alternatively, fault-weakening mechanisms involving fluid transfer propose that dynamic stress ruptures hydraulic seals, facilitating fluid migration and a subsequent pore-pressure increase (*14*, *27*, *28*). Yet, this process relays on particular hydrologic conditions and fails to reconcile with widespread occurrence of delayed triggering beyond volcanic and geothermal settings (*29*). In contrast, cascaded aftershock sequences and creep may offer applicable explanation for delayed triggering across diverse tectonic settings (*6*, *30*–*32*). Still, the physical mechanisms that initiate creep and aftershock cascades remain unclear.

**Fig. 1.**
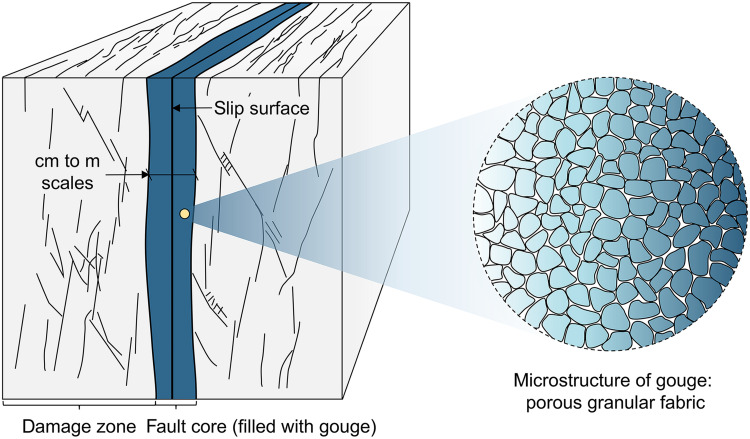
Schematic illustrating the fault zone structure. The slip surface is embedded in gouge layers (centimeter to meter thick), which exhibit a porous granular fabric.

The limited progress in explaining the triggering phenomena mentioned may stem from neglecting the complex mechanical behavior of fault core materials. Remote triggering predominantly occurs in regions with prior seismic activity (*1*, *9*), where the fault core is filled with fault gouge (Fig. 1)—a porous fine-grained material generated by progressive grinding of rock during repeated slip. This gouge layer determines stress distribution along the slip surface, thereby governing earthquake nucleation. As emphasized by Lachenbruch (*33*), achieving a comprehensive understanding of earthquake processes necessitates fundamental knowledge of material properties and mechanical behavior of fault gouge.

Fault gouge features a fine-grained texture, with particle sizes predominantly within the clay-to-silt range (*34*, *35*). Mineralogically, it is rich in clay phases—such as montmorillonite, illite, chlorite, talc, and mixed-layer illite-montmorillonite—with accessory quartz and feldspar (*36*). Such porous granular material displays mechanical behavior more complex than surrounding host rocks. Specifically, high clay content imparts low intrinsic permeability and high compressibility. During shearing, analogous to clay deformation, gouge undergoes dilative or contractive volumetric strain through particle slip and rearrangement (*37*–*40*). Under rapid loading, its low permeability restricts fluid drainage, causing these volumetric strains to generate transient pore-pressure changes that can weaken or strengthen the gouge. Furthermore, the highly ductile nature of gouge deformation leads to sustained residual pore pressure long after loading ceases.

Beyond its high compressibility and ductility, gouge may exhibit stress -path dependence—a behavior inherent to porous granular materials yet seldom considered in fault gouge research, which relies predominantly on monotonic direct-shear tests (*37*). Geotechnical studies have established that such materials display distinct strength and deformation responses under different loading paths in stress space, even at identical loading magnitudes and rates (*41*–*43*). This implies that fault gouge likely responds far more intricately to repeated shear and normal loading imposed by seismic waves than to monotonic shear. In this work, we consider the interaction between gouge and seismic waves and investigate its role in remote triggering.

Here, we present a mechanical model for fault gouge that integrates the plastic deformation of porous granular media under cyclic loading with deformation-pore pressure coupling. Using this model, we develop a computational framework to track stress evolution in gouge-bearing faults during and after remote Rayleigh wave excitation. Our results show that gouge exhibits stress path–dependent pore-pressure generation and dissipation, which govern fault-strength evolution and the onset of triggering. During wave passage, pore pressure can rise far above the perturbation stress amplitude, substantially weakening the fault. This overpressure buildup is dictated by the stress path—compressive loading produces stronger accumulation than shear loading—while wave amplitude, frequency, and duration jointly set its magnitude. Following wave passage, the elevated pore pressure persists, sustaining the weakened state. These findings demonstrate that the pore-pressure response of fault gouge constitutes a fundamental mechanism for remote triggering.

### Dynamic triggering framework

We focus on a planar fault with a simple structure, characterized by the absence of branching, slip surface smoothness, and host-rock homogeneity. The nucleation process over a more complex geometry may hide the effects of gouge response to seismic waves. Our approach is thus designed to unravel the underlying physics governing remote triggering resulting from the intrinsic nature of gouge deformation. In other words, we seek to understand the basic phenomenology first before applying our model to a complex geophysical setting.

**Fig. 2.**
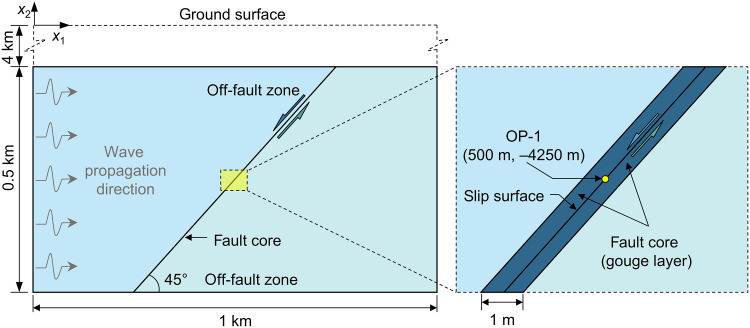
Sketch illustrating the fault geometry, seismic wave propagation, and observation station (OP-1). A two-dimensional dipping normal fault is embedded within a 1-m-thick gouge layer inclined at 45° to the horizontal. The gouge is bounded by an off-fault zone centered at 4.25 km in depth, measuring 1 km in width and 0.5 km in height. Seismic waves propagate horizontally. The observation station OP-1 is located at the center of the slip surface (500 m, −4250 m). *x*_1_ and *x*_2_ denote the horizontal and depth-wise coordinates, respectively.

As remote triggering is observed mainly in extensional regimes (*44*–*46*), we adopt a dipping normal fault—typical in such settings—as our reference configuration (Fig. 2). The fault is centered at 4.25 km in depth with a 45° dip (typically larger than 45°). This minimum dip ensures that Rayleigh waves impose the smallest possible normal stress variation. The fault comprises a 1-m-thick gouge layer (fault core), consistent with geological constraints (*34*, *47*), embedded within a 1 km–by–0.5 km (width × height) off-fault zone. The gouge layer is represented as a poro-elasto-plastic medium, while the off-fault zone is poroelastic. To simulate the perturbation from seismic surface waves (Rayleigh waves), we adopt a quasistatic approach (neglecting inertial effects) whereby a prescribed stress history, derived analytically from the wave’s particle displacements in a homogeneous half-space, is imposed as external loading on the simulation domain.

We implement periodic boundary conditions along the lateral and bottom boundaries of the domain to minimize spurious reactions resulting from simulation domain truncation. The interface between the fault core and the off-fault zone is modeled as partially permeable to circumvent potential misestimations arising from oversimplified end-member assumptions (e.g., fully permeable or impermeable boundaries). We specify the initial stress field of the domain with lithostatic stress. Here, we focus on the prerupture state evolution of faults rather than the rupture process itself and therefore use the classical Mohr-Coulomb criterion to govern the fault strength. To capture the fault stress evolution during and after the perturbation, our simulation comprises two phases: a 200-s wave loading period with the periodic boundary conditions, followed by a switch to static boundaries after the loading ceases.

A constitutive model for fault gouge must capture its inelastic volumetric deformation during compression and shear, as well as its stress path dependence. Critical-state soil mechanics, the modified Cam-Clay (MCC) model, provides a unified framework for these behaviors (*48*). Dynamic rupture simulations adopting the yield criterion within this framework reproduce gouge compaction/dilation, strength degradation, and inelastic strain distributions consistent with geological observations (*49*). However, this model neglects subyield plastic strains, preventing it from capturing accumulated plastic deformation during seismic-cycle loading-unloading. This limitation is addressed by the bounding surface (BS) concept, which allows for plastic strains to develop within the nominal elastic domain (*50*). Here, we use a BS model developed within the critical-state framework to characterize the stress-strain behavior of fault gouge. More details of the dynamic triggering framework are given in Materials and Methods.

## RESULTS

### Stress state evolution during perturbation

A dynamic stress perturbation from a Rayleigh wave, lasting 200 s (10 cycles), is applied to the fault (time history in the Supplementary Materials, fig. S2). We track the temporal evolution of the fault’s stress state—specifically pore pressure ($p_{f\_e}$), shear stress (τ), and effective normal stress ($\sigma_{n}^{\prime }$) on the slip surface—during this loading interval.

**Fig. 3.**
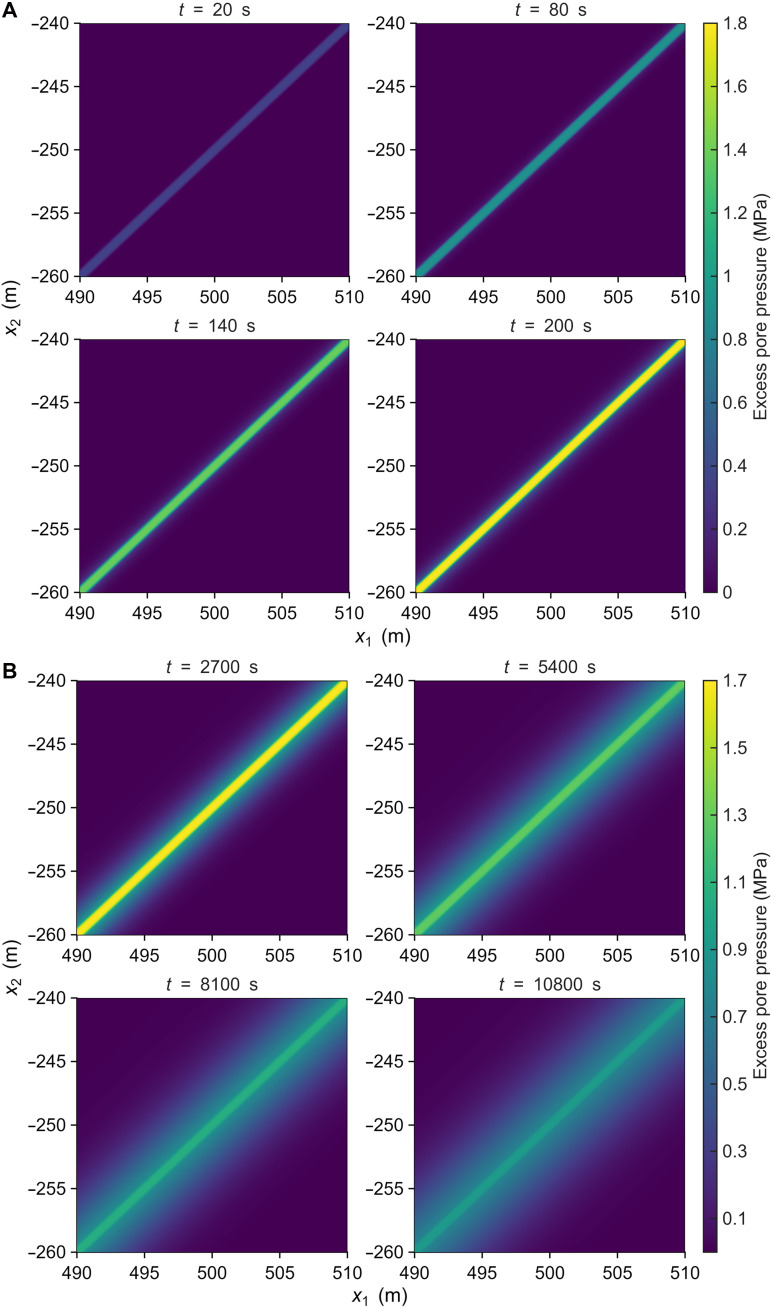
Excess pore-pressure distribution in the fault zone during and after Rayleigh wave perturbation. The color indicates excess pore-pressure magnitude. Panels (**A**) and (**B**) show distributions during perturbation (at 20, 80, 140, and 200 s) and after perturbation (at 2700, 5400, 8100, and 10,800 s), respectively. The excess pore pressure within the fault core increases during the wave passage and subsequently diffuses laterally. *x*_1_ and *x*_2_ represent horizontal and depth-wise coordinates, respectively, consistent with Fig. 2.

Figure 3A shows the distribution of excess pore pressure within the fault zone at four different times during Rayleigh wave loading. By *t* = 20 s, a localized overpressure zone develops within the fault core. Continued dynamic excitation leads to a progressive increase in excess pore pressure. A subtle diffusion-driven excess pore pressure increase is also observed in the immediate off-fault zone adjacent to the core, resulting from fluid seepage; however, the intrinsically low permeability of the gouge limits this effect, confining it spatially and maintaining low magnitudes.

Within the fault core, pore pressure remains spatially uniform. This uniformity arises because the dominant Rayleigh wavelength (*V*_R_ = 3500 m/s, and *T*_R_ = 20 s, so that λ_R_ = 70 km) exceeds the modeled fault dimension (~1 km) by nearly two orders of magnitude, thereby imposing an effectively uniform dynamic stress across the entire zone. As a result, stress-state evolution within the gouge layer is nearly identical at all locations. We therefore track the response at the geometric center of the gouge (point OP-1 in Fig. 2) as a representative in subsequent analyses.

**Fig. 4.**
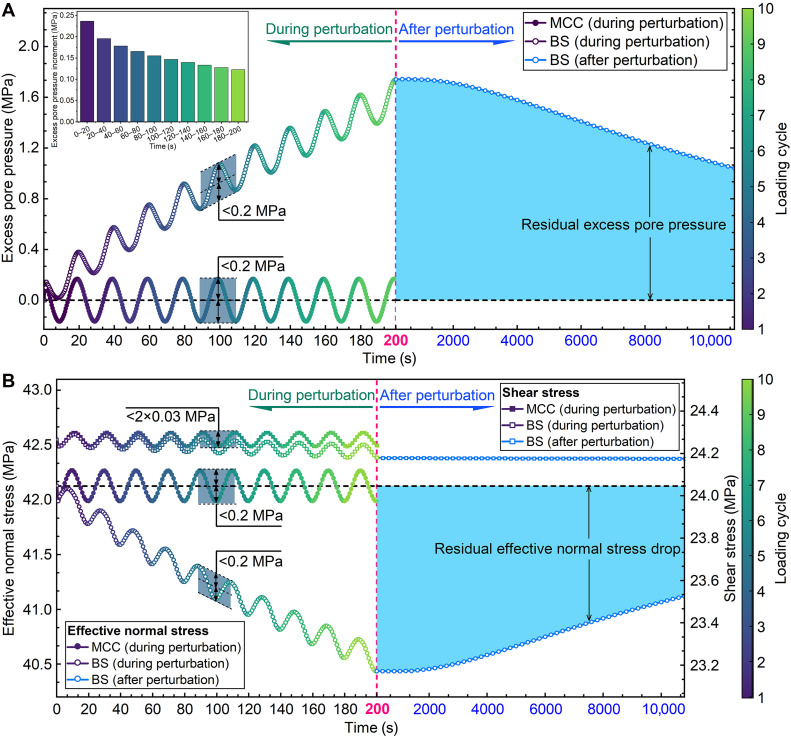
Stress time histories at observation station OP-1 during and after Rayleigh wave perturbation. The station location is shown in Fig. 2. The time histories of excess pore pressure (**A**) and shear and effective normal stresses (**B**) are shown. Lines with open markers represent simulations using the BS model; lines with filled markers correspond to the MCC model. The pink dashed line marks the end of the perturbation phase at 200 s, with colors indicating individual loading cycles. The inset bar graph in (A) highlights the excess pore-pressure increment per loading cycle.

Dynamic stress fluctuations induced by the Rayleigh wave are minor (Fig. 4). On the slip surface, the oscillation amplitudes of pore pressure and effective normal both remain below 0.2 MPa, while shear oscillates by less than 0.03 MPa. These minor perturbations are consistent with observations from remotely triggered events.

Figure 4 also shows that under sustained Rayleigh wave perturbation, the BS simulation reveals a pronounced, stepwise evolution in both pore pressure and stress. After 10 loading cycles, shear stress exhibits a marginal decrease. In contrast, excess pore pressure accumulates by >1.7 MPa, driving a comparable reduction in effective normal stress. This accumulated change is an order of magnitude larger than the instantaneous stress oscillations. Notably, the pore-pressure accumulation rate progressively diminishes with increasing number of loading cycles.

**Fig. 5.**
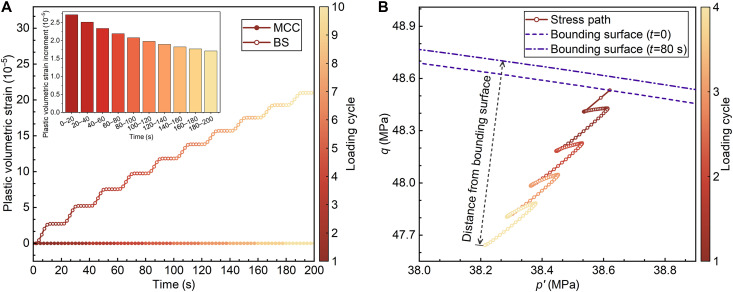
Plastic state evolution of gouge at OP-1 under Rayleigh wave perturbation. (**A**) Time history of plastic volumetric strain. Lines with open and filled circles represent simulations using the BS and MCC models, respectively. The inset panel shows the plastic volumetric strain increment in each loading cycle. (**B**) Stress path in the *q*-*p*′ space. The distance from the stress state (lines with circle markers) to the BS (dashed lines) governs the transition from ductile to brittle behavior. Definitions of *q* and *p*′ are provided in the Supplementary Materials, section S1. Both the strain and the stress path are colored by loading cycle.

To elucidate the mechanism driving the cumulative effect on pore pressure and stress, we compare simulation results from our BS model with a classical elastoplastic model (MCC model) in Figs. 4 and 5. The MCC model is recovered from BS formulation by setting *b* = 1. The definition of *b* is given in the Supplementary Materials, section S1. The essential difference between these frameworks is that the MCC model, consistent with conventional plasticity theory, excludes plastic deformation when the material is close to—but has not yet reached—the nominal yield surface. This simplification holds under monotonic loading because of negligible preyield plastic strains; however, cyclic loading enables these strains to accumulate substantially. As a result of overlooking the buildup of plastic volumetric strain (Fig. 5A), conventional plasticity models fail to replicate the progressive accumulations of pore pressure and stress under Rayleigh wave excitation. Notably, with increasing cycles, the stress state progressively retreats from the nominal yield surface, i.e., BS (Fig. 5B). Increased distance from this surface implies the gradual transition from ductility to brittleness, suppressing plastic strain generation for a given stress increment (Fig. 5A). This explains the slowdown of pore-pressure accumulation.

**Fig. 6.**
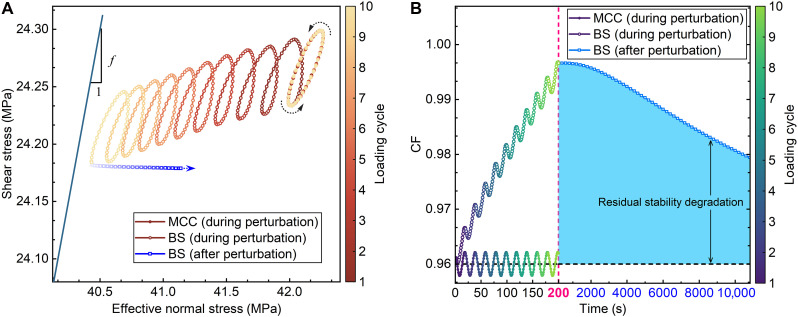
Evolution of the slip state at OP-1 during and after Rayleigh wave perturbation. (**A**) Slip state illustrated by the distance from the stress point to the Mohr-Coulomb failure envelope in the τ-$\sigma_{n}^{\prime }$ space. (**B**) CF, where CF > 1 indicates the onset of slip. Lines with open and filled circles represent BS and MCC model simulations during the perturbation phase, respectively, colored by loading cycle. Lines with square markers indicate evolution after the perturbation.

The evolution of fault stability is captured by the stress-state trajectory in the τ-$\sigma_{n}^{\prime }$ space (Fig. 6A) and by the closeness-to-failure coefficient (CF) (Fig. 6B). As Rayleigh waves propagate through the fault zone, the stress path spirals leftward toward the Mohr-Coulomb failure envelope (τ = *f*$\sigma_{n}^{\prime }$). Yet, after the initial rapid approach, the rate at which the stress path advances toward failure decelerates because of the slowdown of pore-pressure buildup. In addition, CF—defined as the ratio of shear stress to shear resistance on the slip surface—rises from 0.96 to more than 0.995 by the end of the Rayleigh wave perturbation. Were the wave train to persist only marginally longer or the fault initial state to be slightly closer to failure (e.g., CF_0_ = 0.965), slip initiation would occur during the wave passage. By contrast, in simulations using the MCC model, the stress state follows a closed, fixed elliptical loop in the τ-$\sigma_{n}^{\prime }$ space, and CF remains essentially anchored near its starting value, oscillating by just ∼0.002. Such limited fluctuation fails to reproduce slip onset on the subcritically stressed fault.

### Stress state evolution after perturbation

Building on the Rayleigh wave perturbation simulations in the previous section, we next explore the evolution of the fault’s stress state over 3 hours following wave passage and assess its impact on fault instability, with the aim of clarifying the drivers of delayed triggering. During the postperturbation interval, pore-fluid migration drives a systematic redistribution of pore pressure throughout the fault zone (Fig. 3B). As excess pore pressure decays within the core, it diffuses laterally into the surrounding host rock, gradually elevating pore pressure over a roughly 10-m-wide region around the fault core. To further characterize the postperturbation evolution of the stress state on the slip plane, we use the central point of the surface (OP-1) as a representative location for subsequent analyses.

Following the passage of the Rayleigh wave, the gouge layer undergoes progressive consolidation. Excess pore pressure along the slip interface dissipates because of drainage at the fault core boundary, declining from ~1.7 to ~1.0 MPa in 3 hours (Fig. 4A). Critically, dissipation initiates only after a delay of ∼30 min. This temporal offset is attributed to the evolution of the internal hydraulic gradient. Immediately after cessation of the perturbation, pore pressure remains near-uniform within the fault core. The steep hydraulic gradient at the boundary requires considerable time to propagate inward, thereby providing sufficient hydraulic driving force for fluid drainage in central regions.

With pore pressure dissipating, the effective normal stress on the sliding surface undergoes gradual recovery (Fig. 4B). It remains below 40.5 MPa during the first 30 min, followed by an increase of ~0.7 MPa over the subsequent 2.5 hours. Crucially, the effective normal stress remained substantially below its preperturbation level even after 3 hours of relaxation. This weakened state is expected to persist over an extended temporal duration because of the intrinsically low permeability of the gouge layer. In contrast, shear stress on the slip surface remains constant throughout the postperturbation period (Fig. 4B).

As effective normal stress recovers, the stress state on the slip surface translates horizontally to the right in the τ-$\sigma_{n}^{\prime }$ space, progressively distancing itself from the Mohr-Coulomb failure envelope (Fig. 4E). Crucially, this retreat decelerates over time, owing to the diminishing hydraulic gradient across the gouge layer during pore-pressure re-equilibration, which in turn slows internal seepage. The CF hovers near 0.997 for roughly 30 min before gradually declining; after 2.5 hours, it has fallen by only ~0.017, remaining markedly above its preperturbation level (Fig. 4F). In other words, the fault remains in nearly critical state for several hours following Rayleigh wave passage, inhibiting a rapid return to its original stable configuration.

### Contributions of seismic wave characteristics

The preceding sections demonstrated that Rayleigh waves can induce pronounced elevation in pore pressure, thereby facilitating fault weakening. Here, we delve further into how specific wave characteristics—namely amplitude, frequency, and the partitioning between compressional and shear components—influence the pore-pressure response.

**Fig. 7.**
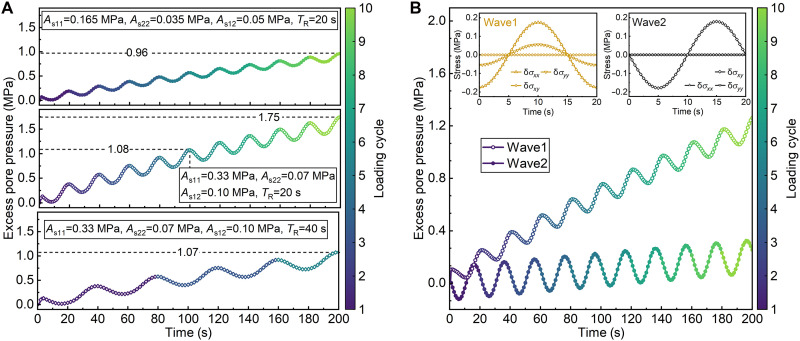
Pore-pressure responses at OP-1 under different types of Rayleigh wave trains. Responses are colored by loading cycle. (**A**) Simulations under varying amplitude (upper and middle panels) and frequency (middle and lower panels). *A*_s*ij*_ and *T*_R_ denote the amplitude of dynamic stress components and the Rayleigh wave period, respectively. (**B**) Lines with open and filled circles represent simulations under pure compressional (Wave1) and pure shear (Wave2) components, respectively. The corresponding dynamic stress (${\delta \sigma }_{\mathrm{ij}}$) are shown in the top insets.

To isolate the effects of amplitude and frequency, we generated three Rayleigh wave trains with different dynamic stress amplitudes (*A*_s*ij*_, where *i* and *j* are stress-component indices) and periods (*T*_R_) (Fig. 7A). The first wave train (upper panel) has amplitudes exactly half those of the second train (middle panel). As anticipated, the total pore-pressure increase over an identical duration is lower for the first train. Notably, however, its cumulative increase of 0.96 MPa exceeds half of the second train’s 0.87 MPa, revealing a clear nonlinear relationship between wave amplitude and pore-pressure buildup. This nonlinearity is consistent with the stress path effects illustrated in Fig. 4D: Higher amplitudes drive the stress state farther from the nominal yield surface, reducing plastic deformation per unit stress increment and thereby lowering the pore-pressure accumulation efficiency.

Next, a comparison between the second and third wave trains (lower panel) highlights that over an equivalent time window, higher-frequency waves generate greater cumulative pore pressure. Crucially, when normalized by the number of loading cycles, both achieve identical pore-pressure growth—1.08 MPa after five cycles—confirming that the apparent time-dependent enhancement simply reflects an increased cycle count rather than a distinct frequency effect.

Last, we decompose Rayleigh waves into their pure compressional and shear components by tuning coefficients *C*_1R_-*C*_4R_ [Wave1 and Wave2 in Fig. 7B]. These coefficients are defined in the Supplementary Materials, section S1. Both waves are scaled to comparable dynamic stress amplitude. While the shear-only wave (Wave2) produces pore-pressure oscillations comparable to the compressional wave (Wave1), its contribution to net pore-pressure buildup is negligible. This unequivocally identifies the compressional component as the principal driver of pore-pressure accumulation under Rayleigh wave excitation.

### Parameter analysis

As a novel application of the BS plasticity framework to fault gouge, direct experimental constraints on key plastic parameters (*H*_0_ and λ) remain scare. Considering this, we conduct a systematic parameter sensitivity analysis in this section. The details of these parameters are given in the Supplementary Materials, section S1.

**Fig. 8.**
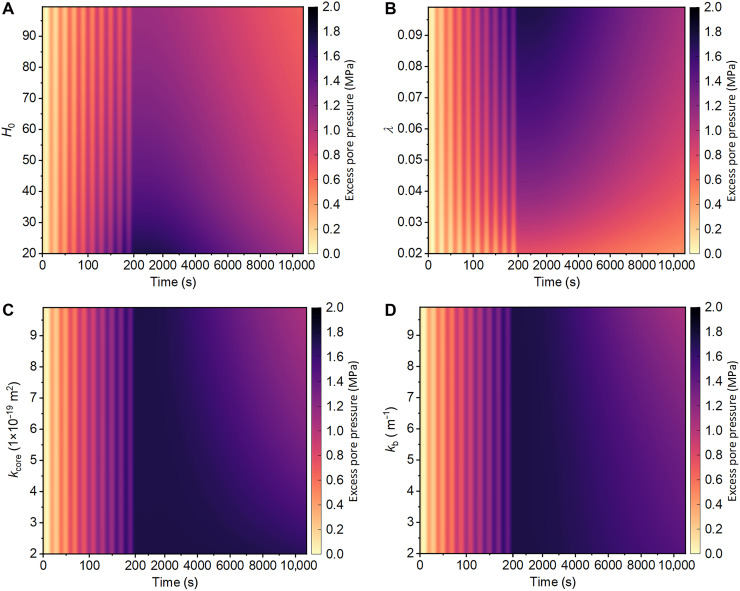
Influence of key model parameters on excess pore pressure at OP-1 during Rayleigh wave perturbation. (**A** and **B**) Effects of plastic parameters *H*_0_ and λ. (**C** and **D**) Effects of permeability parameters *k*_core_ and *k*_b_. *H*_0_ controls the interpolation of the plastic modulus within the BS; λ controls the plastic modulus on the BS. *k*_core_ is the permeability of the fault gouge, and *k*_b_ governs the permeability at the gouge layer boundary.

Figure 8 (A and B) illustrates the respective impacts of *H*_0_ (controlling the plastic modulus interpolation within the yield surface) and λ (controlling the plastic modulus) on slip-surface pore-pressure evolution. Increasing *H*_0_ or decreasing λ elevates the plastic modulus, thereby reducing pore-pressure accumulation—consistent with the behavior observed in Fig. 4. Critically, while the magnitude of pore-pressure change varies with these parameters, the fundamental evolution characteristics remain robust: Pore-pressure accumulation decelerates, and dissipation exhibits persistent delay.

Figure 8 (C and D) analyzes the impacts of gouge permeability and gouge layer boundary permeability. During seismic perturbation, the slip-surface pore-pressure response is insensitive to permeability variations. The fault core behaves as effectively undrained when gouge permeability is sufficiently low. In contrast, during consolidation, even modest reductions in permeability markedly retard pore-pressure dissipation. This highlights gouge layer permeability as a critical control on the recovery of fault strength following seismic wave passage.

## DISCUSSION

### Role of fault gouge

Dynamic stresses carried by teleseismic waves are negligible compared to ambient stresses. The perturbation amplitude of surface waves responsible for remote triggering is typically only 0.01 to 1 MPa—two to three orders of magnitude lower than the shear strength of faults at seismogenic depths (*20*, *51*). According to the Coulomb failure criterion, such minuscule perturbations can only induce slip on faults already at the brink of failure. The prevailing hypothesis thus invokes a subset of faults in the late stages of their seismic cycle (*6*, *52*). However, as fault populations within a system are uniformly distributed across all stages of the cycle, the fraction of such critically stressed faults is vanishingly small. This poses a clear contradiction with the unexpectedly high incidence of remote triggering revealed by statistical analyses (*53*).

The poro-elasto-plastic response of fault gouge to seismic wave perturbations offers a resolution. Our results (Fig. 4) show that Rayleigh wave excitation produces excess pore-pressure accumulation within the gouge layer. Over minutes, this accumulation can raise to 1.7 MPa—more than 10 times the direct Coulomb stress fluctuation (${|\delta \tau -f\delta \sigma_{n}^{\prime }|}_{\text{max}}$ ≈ 0.15 MPa)—markedly reducing the fault strength. This mechanism implies that even low-amplitude Rayleigh waves can trigger slip on subcritical faults. Given the ubiquity of fault gouge, cyclical pore-pressure buildup provides a robust and universal explanation for the high incidence of transient remote triggering across diverse tectonic settings.

The pore-pressure increase results from the contraction of fault gouge. Fault gouge can also dilate, leading to a pore-pressure decrease. Under high shear stress ratios, which behavior occurs depends on the consolidation (or density) state, as demonstrated in the fault gouge shear experiments by Morrow and Byerlee (*54*). Natural fault gouge is typically clay-rich and therefore predominantly contractive (*36*, *54*). However, under extreme overconsolidation—conditions that are probably uncommon in natural faults—dilatancy may develop, resulting in fault strengthening (*55*, *56*). This may explain the observed decrease in seismicity rates following the passage of remote seismic waves (*57*, *58*).

Because of the current lack of cyclic-loading experiments on natural fault gouge, the constitutive parameters are drawn from representative geotechnical practice. Accordingly, the rheology is intended as a phenomenological framework to explore mechanical behavior, rather than a quantitatively constrained representation of seismogenic fault materials.

### Role of seismic waves

Building on our previous findings, pore-pressure buildup in fault gouge drives remote triggering by weakening the fault. Figure 7A shows that this overpressure depends on the wave’s amplitude, frequency, and duration: Wave trains containing more high-amplitude pulses produce larger pore-pressure increases. Consequently, wave trains consisting of only a few large pulses embedded within low-amplitude oscillations are less effective at triggering slip compared to those sustaining moderate amplitudes. This insight explains why field studies consistently fail to correlate peak ground displacement with remote triggering rates (*14*, *18*).

Hill (*20*) reported that Love waves, compared with Rayleigh waves of identical amplitude and period, more readily induce Coulomb failure on vertical strike-slip faults. Given the prevalence of such structures in tectonic regimes, one might consequently expect a surge of triggers coincident with Love wave arrivals. However, seismic catalogs show that extensive triggered events align with the later-arriving Rayleigh waves (*1*, *59*), implying that Rayleigh waves exert a stronger influence in remote triggering. Unlike purely shear Love waves, Rayleigh waves combine compressional and shear motion. Our results show that their compressional component generates considerable pore-pressure accumulation, weakening the fault more effectively than the shear stress from equal-amplitude Love waves. This explains the superior triggering efficiency of Rayleigh waves. However, Love waves may dominate under conditions including the following: (i) immature faults, where underdeveloped gouge limits pore-pressure buildup; (ii) stronger Love wave amplitude, where higher shear stress outweighs strength reduction from Rayleigh waves (*60*, *61*).

The enhanced efficacy of compressional waves reflects their propensity to impose stress paths that drive irreversible volumetric deformation in fault gouge. Consequently, assessing a teleseismic wave’s triggering potential transcends conventional metrics like amplitude, frequency, and duration; it necessitates a detailed reconstruction of the stress trajectory at the nucleation depth. This insight resolves the puzzle of why empirical correlations between surface wave parameters and triggering rates remain elusive. Future research should, therefore, prioritize techniques to invert surface seismograms for stress paths within the fault zone, enabling a comprehensive evaluation of remote triggering potential.

### Mechanism of delayed dynamic triggering

Delayed triggering—characterized by a rise in seismicity minutes to months after seismic wave passage—remains poorly explained. Our findings reveal (Fig. 4) that transient perturbations from seismic waves induce enduring stress alterations. The accumulated pore pressure, generated during seismic wave transit, dissipates slowly because of the low permeability of fault gouge, maintaining the fault in a failure-prone state long after the perturbation ceases. This sustained weakening lays the groundwork for the postwave increase in seismicity.

The triggering delay spans a wide timescale—from minutes to months—which depends on two factors: the degree of fault weakening and the nature of postseismic perturbations. Greater pore-pressure accumulation brings the fault closer to failure, favoring shorter delays. Aftershock cascades can induce short- to medium-term delays through cumulative stress changes, whereas slow tectonic loading may lead to long-term delays even in critically stressed faults.

The delayed triggering mechanism hinges on the dissipation of pore pressure within fault gouge. Lower permeability slows dissipation, favoring delayed triggering. Given that the dissipation rate scales inversely with permeability, an order-of-magnitude change in permeability can cause a 10-fold shift in dissipation time—providing a key basis for the wide range of observed delays. This mechanism is particularly relevant in volcanic and geothermal settings, where low-permeability, fluid-rich fault gouge is expected, as demonstrated by Lupi *et al.* (*62*) who observed delayed activity in the Campi Flegrei caldera following seismic events.

### Implications on tectonic sensitivity in dynamic triggering

Although a dipping normal fault is used as the reference setup, the proposed pore-pressure buildup mechanism applies to various fault geometries, as it depends on the stress path in the *p*-*q* space (Fig. 5B), not on the variation of stresses resolved on the fault plane. This suggests that the angle between the seismic wave direction and the fault plane has little effect on pore-pressure accumulation. However, remote triggering is still influenced by fault geometry because, besides pore-pressure buildup, seismic waves also directly induce stress fluctuations on the fault. These two mechanisms jointly govern triggering. Rayleigh wave triggering, dominated by pore-pressure buildup, is thus less geometry-sensitive. In contrast, Love wave triggering depends more strongly on geometry (favoring strike-slip faults) because of its weaker pore-pressure effect. Consequently, the higher triggering efficiency of Rayleigh waves tends to be more pronounced on normal or reverse faults.

Remote triggering is observed predominantly in extensional tectonic settings (compared to compressional ones) (*44*–*46*), which are characterized by dipping normal faults and transtensional strike-slip faults. This preference is difficult to explain by fault geometry (i.e., fault orientation). Instead, it may relate to factors like ambient stress levels and fluid conditions—key differences between these tectonic settings. Our poro-elasto-plastic theoretical framework, which can incorporate these factors, may promote our understanding of the tectonic sensitivity of remote triggering.

## MATERIALS AND METHODS

A poro-elasto-plastic model was developed to characterize the cyclic loading behavior of fault gouge. This framework incorporates a BS plasticity concept rooted in critical-state soil mechanics to capture elastoplastic deformation while coupling solid matrix deformation with pore-pressure evolution through Biot’s theory and Darcy’s law. Detailed governing equations and simulation parameters are provided in the Supplementary Materials, section S1. Numerical implementation was achieved using COMSOL Multiphysics, leveraging its Structural Mechanics module and Mathematics interface to establish our physics. Model validation against the literature (*63*) is presented in the Supplementary Materials, section S2. Additional specifications regarding the dynamic triggering framework—including seismic wave perturbations, boundary and initial conditions, and fault strength criterion—appear in the Supplementary Materials, section S1.

## Supplementary Materials

### This PDF file includes:

Supplementary Text Figs. S1 to S4 Tables S1 to S4 References
