# Supplementary material for: What triggers seismicity thousands of kilometers away from a mainshock?
Source: Sci Adv. 2026 Jun 24;12(26):eaec4754. doi: 10.1126/sciadv.aec4754 (PMC13292997; doi:10.1126/sciadv.aec4754)
Supplement: Supplementary file 1 — Supplementary Text Figs. S1 to S4 Tables S1 to S4 References [file sciadv.aec4754_sm.pdf]

Science Advances
AAAS

Supplementary Materials for

What triggers seismicity thousands of kilometers away from a mainshock?

Chao Huang and Jun Yang

Corresponding author: Jun Yang, junyang@hku.hk

Sci. Adv. **12**, eaec4754 (2026)
DOI: 10.1126/sciadv.aec4754

This PDF file includes:

Supplementary Text
Figs. S1 to S4
Tables S1 to S4
References

Supplementary Text

S1. Dynamic Triggering framework Setup

Governing equations

We model the gouge layer as a poro-elasto-plastic medium and the surrounding off-fault zone as a poroelastic medium. The governing equations for momentum and fluid conservation in fluid-saturated porous media are derived by integrating Biot's theory with Darcy's law. These coupled equations describe the interaction between porous media deformation and pore pressure, expressed as

Momentum balance:

$$\nabla \cdot \left(\sigma'_{ij} + \alpha p_f \delta_{ij} \right) = f_i \tag{1}$$

Mass conservation:

$$\rho_f S \frac{\partial p_f}{\partial t} - \rho_f \alpha \frac{\partial \varepsilon_v}{\partial t} - \nabla \cdot \left(\rho_f \frac{k}{\eta_f} \nabla p_f \right) = 0 \tag{2}$$

In these expressions, $\sigma_{ij} = \sigma'_{ij} + \alpha \delta_{ij} p_f$ donates the total stress, decomposed into effective stress σ'_{ij} and pore fluid pressure p_f. f_i is a body force, corresponding here to the dynamic stress induced by seismic waves. α and k are Biot's coefficient and intrinsic permeability of porous medium, respectively. Subscripts "core" and "off" distinguish parameters for the fault core (gouge layer) and the off-fault zone, e.g., α_{core} and α_{off}. η_f and ρ_f donate fluid dynamic viscosity and fluid density. The volumetric strain is given by $\varepsilon_v = \varepsilon_{kk}$. The storage coefficient $S = \phi / K_f$, depending on porosity ϕ and fluid bulk modulus K_f. Note that the sign convention for the porous media takes compression as positive. The porous parameters of fault gouge and off-fault rock are shown in Table S1 and Table S2, respectively, and the pore fluid parameters are listed in Table S3, consistent with those in references (*64*) and (*65*).

Assuming the off-fault zone behaves as a linear elastic medium, its constitutive relationship is expressed as

$$\sigma_{ij} = K_{\text{off}} \varepsilon_v \delta_{ij} + 2G_{\text{off}} \left(\varepsilon_{ij} - \frac{1}{3} \varepsilon_v \delta_{ij} \right) \tag{3}$$

where the bulk modulus K_{off} and shear modulus G_{off} are calculated from P-wave velocity V_P, S-wave velocity V_S, and density ρ_{off}, with $G_{\text{off}} = \rho_{\text{off}} V_S^2$, $K_{\text{off}} = \rho_{\text{off}} \left(V_P^2 - 4/3 V_S^2 \right)$. Material parameters of the off-fault zone rock (Table S2) are consistent with those in reference (*20*).

We employ a bounding surface (BS) model grounded in critical-state soil mechanic to describe the stress-strain behavior of fault gouge. The BS model is formulated in q-p' stress space, where p' denotes the effective mean normal stress and q the deviatoric stress, defined as

$$p' = \frac{\sigma'_{kk}}{3} \tag{4}$$

$$q = \sqrt{\frac{3}{2} s_{ij} s_{ij}}, \quad s_{ij} = \sigma'_{ij} - p' \delta_{ij} \tag{5}$$

Total strain increment $\mathrm{d}\varepsilon_{ij}$ is decomposed additively into elastic $\mathrm{d}\varepsilon_{ij}^e$ and plastic $\mathrm{d}\varepsilon_{ij}^p$ components

$$\mathrm{d}\varepsilon_{ij} = \mathrm{d}\varepsilon_{ij}^e + \mathrm{d}\varepsilon_{ij}^p \tag{6}$$

The response associated with elastic part, similar to Eq. (3), is governed by the elastic bulk modulus K^e_{core} and shear modulus G^e_{core}, determined by critical-state theory as

$$K^e_{\text{core}} = \frac{1+e_0}{\kappa} p', \; G^e_{\text{core}} = \frac{3\left(1-2\upsilon_{\text{core}}\right)}{\left(1+\upsilon_{\text{core}}\right)} K^e_{\text{core}} \tag{7}$$

where υ_{core} is the Poisson's ratio of the fault core, e_0 the initial void ratio, and κ the slop of the swelling line in e-ln(p') plane (Fig. S1a).

The bounding surface, playing a similar role to the yield surface in classical plastic theory, is represented by an ellipse in p'-q stress space (Fig. S1b) expressed as

$$\bar{F}\left(\bar{\sigma}'_{ij}, \bar{p}_c\right) = \bar{p}' + \frac{\bar{q}^2}{M_c^2 \bar{p}'} - \bar{p}_c = 0 \tag{8}$$

where variables with bars "-" lie on the bounding surface, M_c is the stress ratio (q/p') at critical state (Fig. S1b), and $\bar{p}_c$ controls the bounding surface size, analogous to yield stress.

To define the image stress $\bar{\sigma}'_{ij}$ (the stress on the bounding surface), the current stress σ'_{ij} is radially projected to the surface using the origin of stress space as the projection center. This mapping rule can be mathematically expressed as

$$\bar{\sigma}'_{ij} = b\sigma'_{ij} \tag{9}$$

The assumption of Eq. (9) implies the loading surface (i.e., $F(\sigma_{ij}, p_c)$ in Fig. S1b), which passes through the current stress, is homothetic to the bounding surface with the projection center as the center of homothecy. Independent variable b can be further interpreted as the similarity ratio of the bounding and loading surfaces.

The initial similarity ratio, b_0 corresponding to the over consolidation ratio (OCR), defined as the ratio of the maximum past stress state to current stress. The evolution of b is derived from consistent condition (4), expressed as

$$\mathrm{d}b = \mathrm{d}\bar{p}_c \left(b^2 K^p_{\text{core}} - b\bar{K}^p_{\text{core}}\right) / \left(\bar{p}_c \bar{K}^p_{\text{core}}\right) \tag{10}$$

where K^p_{core} is the plastic modulus at the current stress state; $\bar{K}^p_{\text{core}}$ is the image plastic modulus on the bounding surface.

Plastic strain rate in accordance with associated flow rule is determined as

$$\mathrm{d}\varepsilon^p_{ij} = \langle \Lambda \rangle R_{ij}, \; R_{ij} = L_{ij} = \frac{\partial \bar{F}}{\partial \bar{\sigma}'_{ij}} \tag{11}$$

where the symbol ⟨⟩ stands for Macauly brackets such that $\langle x \rangle = x$ if $x \geq 0$ and $\langle x \rangle = 0$ if $x < 0$. R_{ij} donates the direction of plastic flow, aligning with the gradient direction of bounding surface L_{ij} (i.e., **L** in Fig. S1b) according to associate flow rule. Plastic multiplier Λ can be defined as follows

$$\Lambda = \frac{1}{K^p_{\text{core}}} \frac{\partial F}{\partial \sigma'_{ij}} \mathrm{d}\sigma'_{ij} = \frac{1}{\bar{K}^p_{\text{core}}} \frac{\partial \bar{F}}{\partial \bar{\sigma}'_{ij}} \mathrm{d}\bar{\sigma}'_{ij} \tag{12}$$

In accordance with critical state theory, isotropic hardening related to the plastic volumetric strain rate $\mathrm{d}\varepsilon^p_v$ governs the evaluation of the yield stress $\bar{p}_c$

$$\mathrm{d}\bar{p}_c = \bar{p}_c \frac{1+e_0}{\lambda - \kappa} \mathrm{d}\varepsilon^p_v \tag{13}$$

where λ is the slope of the normal compression line in e-ln(p') plane (Fig. S1a), governing the plastic modulus on the bounding surface.

$\bar{K}^p_{\text{core}}$ is obtained by imposing the consistent condition on the bounding surface, and K^p_{core} is determined by $\bar{K}^p_{\text{core}}$ and an additional plastic modulus that is proportional to the similarity ratio b. They are given as

$$\bar{K}^p_{\text{core}} = \frac{4M_c^2}{9}\frac{1+e_0}{\lambda-\kappa}\bar{p}\left(2\bar{p}-\bar{p}_c\right)\bar{p}_c \tag{14}$$

$$K^p_{\text{core}} = \bar{K}^p_{\text{core}} + H_0\frac{16M_c^2}{9}\frac{1+e_0}{\lambda-\kappa}\bar{p}^3\left(\frac{b-1}{b}\right) \tag{15}$$

where H_0 is a parameter governing the interpolation of the plastic modulus within the bounding surface.

The algorithmic implementation of the bounding surface plasticity framework is detailed in S3. As the novel application of a bounding surface model to fault gouge, parameter selection lacks established constraints; thus, values standard in geotechnical practice are adopted (Table S1).

Seismic Wave Perturbation

We modeled fault-zone stress perturbations produced by the dynamic stress, $\delta\sigma_{ij}(t)$, from a passing fundamental-mode Rayleigh wave. In simulations, $\delta\sigma_{ij}(t)$ was converted to equivalent external loads via Gaussian integration and applied to the fault zone.

The Rayleigh waves related particle displacements in a homogeneous half-space are given by (*20*)

Horizontal displacement:

$$u_1\left(x_1,x_2,t\right) = -A_\text{R}k_\text{R}\sin\left(\phi\right)\left[\exp\left(C_{1\text{R}}k_\text{R}x_2\right) - C_{2\text{R}}\exp\left(C_{3\text{R}}k_\text{R}x_2\right)\right] \tag{16}$$

Vertical displacement:

$$u_2\left(x_1,x_2,t\right) = A_\text{R}k_\text{R}\cos\left(\phi\right)\left[C_{1\text{R}}\exp\left(C_{1\text{R}}k_\text{R}x_2\right) - C_{4\text{R}}\exp\left(C_{3\text{R}}k_\text{R}x_2\right)\right] \tag{17}$$

where $\phi = k_R x_1 - \omega t$ is phase, A_R is amplitude, ω=2π/T_R is angular frequency, k_R=2π/($V_\text{R}\cdot T_\text{R}$) is wavenumber, T_R is wave period, V_R is Rayleigh velocity and $C_{1\text{R}}$-$C_{4\text{R}}$ are decay coefficients.

$C_{1\text{R}}$ and $C_{3\text{R}}$ are defined by

$$C_{1\text{R}} = \sqrt{1-\left(\frac{V_\text{R}}{V_\text{P}}\right)^2} \tag{18}$$

$$C_{3\text{R}} = \sqrt{1-\left(\frac{V_\text{R}}{V_\text{S}}\right)^2} \tag{19}$$

$C_{2\text{R}}$ and $C_{4\text{R}}$ follow from the free-stress boundary conditions ($\delta\sigma_{22}$=0 and $\delta\sigma_{12}$=0) at the surface (x_2=0).

The corresponding strain components are

$$\varepsilon_{11} = u_{1,1} = -A_R k_R^2\cos\left(\phi\right)\left[\exp\left(C_{1R}k_R x_2\right) - C_{2R}\exp\left(C_{3R}k_R x_2\right)\right] \tag{20}$$

$$\varepsilon_{22} = u_{2,2} = A_R k_R^2\cos\left(\phi\right)\left[C_{1R}^2\exp\left(C_{1R}k_R x_2\right) - C_{3R}C_{4R}\exp\left(C_{3R}k_R x_2\right)\right] \tag{21}$$

$$\begin{aligned}\varepsilon_{12} &= \frac{1}{2}\left(u_{1,2}+u_{1,2}\right)\\ &= -\frac{1}{2}A_R k_R^2\sin\left(\phi\right)\left[2C_{1R}\exp\left(C_{1R}k_R x_2\right) - \left(C_{2R}C_{3R}+C_{4R}\right)\exp\left(C_{3R}k_R x_2\right)\right]\end{aligned} \tag{22}$$

where $u_{i,j}$ represents the partial derivative of u_i with respect to x_j.

Dynamic stresses are then computed from

$$\delta\sigma_{ij} = \lambda_{\text{off}}\delta_{ij}\varepsilon_{kk} + 2\mu_{\text{off}}\varepsilon_{ij} \tag{23}$$

where $\mu_{\text{off}} = \rho_{\text{off}} V_S^2$ and $\lambda_{\text{off}} = \rho_{\text{off}}\left(V_P^2 - 2V_S^2\right)$ are Lame constants in the off-fault zone.

Wave parameters (Table S4) follow Hill (*20*). Because wavelength of distant Rayleigh waves (10s of km) exceeds the model size (~ 1 km) by orders of magnitude, the dynamic stress is nearly uniform across the domain. Unless otherwise stated, the dynamic stress time history employed in this study is shown in Fig. S2.

To avoid boundary-induced artifacts from fixed-displacement or roller constraints, we applied periodic boundary conditions (Eqs. (16) and (17)) to the model's lateral and basal boundaries.

Initial Stress field

We specify the initial stress of the fault with lithostatic stress, defined as

$$\sigma'_{22\_\text{ini}} = \left(\rho_{\text{off}} - \rho_f\right) gh \tag{24}$$

$$\sigma'_{11\_\text{ini}} = \frac{\upsilon_{\text{off}}}{1-\upsilon_{\text{off}}}\sigma'_{22\_\text{ini}} \tag{25}$$

where σ'_{11_ini} and σ'_{22_ini} are the initial effective stress components along the x_1 and x_2 directions (Fig. 2); ρ_{off} and ρ_f are the densities of the off-fault zone and pore fluid; υ_{off} is the Poisson's ratio of the off-fault zone; g is gravitational acceleration; h is depth below free surface.

Partially Permeable Interface

Permeability across the interface between the fault core and surrounding zone can strongly influence pore-pressure evolution in the core. To avoid oversimplified end-member assumptions (fully permeable or impermeable), we treat the interface as partially permeable, applying a Robin boundary condition

$$\nabla p_f \cdot \mathbf{n}_{\text{b}} = k_{\text{b}}\left(p_f - p_{f\_\text{b}}\right) \tag{26}$$

where $\mathbf{n}_{\text{b}}$ and p_{f_b} are the unit normal vector and pore pressure at the boundary, respectively; k_{b} is an empirical parameter governing boundary permeability. As k_{b} approaches zero, the boundary becomes impermeable; as k_{b} tends to infinity, it becomes fully permeable. The value of k_{b} is 10 m^{-1}.

Failure criteria

The frictional behavior of faults during rupturing is well characterized by the rate-and-state friction (RSF) model. However, this study focuses on the pre-rupture state evolution of faults rather than the rupture process itself and therefore employs the Mohr-Coulomb criterion to represent the fault failure behavior. The fault shear strength is governed by

$$\tau_{\text{c}} = f\sigma'_{\text{n}} = f\left(\sigma_{\text{n}} - p_f\right) \tag{27}$$

where f is the friction coefficient; τ_{c} is the critical shear stress on the fault; σ_{n} and σ'_{n} are the total and effective normal stress on the fault; $p_f = p_{f\_\text{ini}} + p_{f\_\text{e}}$ is on-fault pore pressure; p_{f_ini} is the initial pore pressure used to adjust the initial strength on the fault; p_{f_e} is the excess pore pressure generated by the passing of Rayleigh waves.

To quantify slip state, a closeness-to-failure coefficient (*CF*) is introduced, defined as the ratio of shear stress to critical shear stress on the fault plane

$$CF = \frac{\tau}{\tau_{\text{c}}} = \frac{\tau}{f\left(\sigma_{\text{n}} - p_{f\_\text{ini}} - p_{f\_\text{e}}\right)} \tag{28}$$

where the initial pore pressure p_{f_ini} can be determined by the initial closeness-to-failure coefficient CF_0. The values of f and CF_0 are 0.6 and 0.96, respectively.

S2. BS Model Verification

To ensure the reliability of our numerical framework, we perform a validation exercise focused on the verification of the fault gouge stress-strain behavior governed by the bounding surface model. The stress-strain behavior is validated by simulating an undrained triaxial test on a single element. We adopt identical material parameters to those used by Manzari and Nour (*63*) and compare the simulated stress-strain curves and stress paths with their reference results. The shear stress versus vertical strain (Fig. S3a) and the evolution of mean versus deviatoric stress (Fig. S3b) are essentially identical between the two datasets, indicating that our implementation accurately reproduces the constitutive response of the bounding surface model.

S3. Algorithm for BS Model

The return mapping algorithm is used for stress update in bounding surface plasticity framework, which involves an elastic predictor phase and a plastic corrector phase, as illustrated in Fig. S4. At the beginning of each load increment, a trial stress on the loading surface is calculated by assuming a fully elastic incremental strain. Projecting the trail stress on the bounding surface by the mapping rule, an image trail stress is obtained. If the image trial stress violates the yield criteria, a plastic correction step then will correct it back on the bounding surface. The details of the method are described in Box S1.

Box S1. Stress Updates Algorithm

Input: states at the end of the n step (${}^{(n)}\sigma'_{ij}$, ${}^{(n)}\bar{p}_c$, ${}^{(n)}b$) and strain increment for the n+1 step ${}^{(n+1)}\Delta\varepsilon_{ij}$.

Output: states at the end of the n+1 step (${}^{(n+1)}\sigma'_{ij}$, ${}^{(n+1)}\bar{p}_c$, ${}^{(n+1)}b$).

Step-1: elastic predictor. Calculate the elastic stiffness matrix and trial stress.

$$D^e_{ijkl} = D^e_{ijkl}\left({}^{(n)}\sigma'_{ij}\right) \tag{B1}$$

$${}^{(n+1)}\sigma'_{ij,\,\text{trial}} = {}^{(n)}\sigma' + D^e_{ijkl}\,{}^{(n+1)}\Delta\varepsilon_{ij};\ {}^{(n+1)}\bar{\sigma}'_{ij,\,\text{trial}} = {}^{(n)}b\,{}^{(n+1)}\sigma'_{ij,\,\text{trial}} \tag{B2}$$

Step-2: check yield condition.

$$\begin{cases} \text{material remains elastic, go to step 3} & \text{if } \bar{F}\left({}^{(n+1)}\bar{\sigma}'_{ij,\,\text{trial}},\ {}^{(n)}\bar{p}_c\right) \le 0 \\ \text{plastic yielding occurs, go to step 4} & \text{if } \bar{F}\left({}^{(n+1)}\bar{\sigma}'_{ij,\,\text{trial}},\ {}^{(n)}\bar{p}_c\right) > 0 \end{cases}$$

Step-3: update states. Update variables ${}^{(n+1)}\bar{\sigma}'_{ij}$, ${}^{(n+1)}\bar{p}_c$, and ${}^{(n+1)}b$ for the n+1 step.

$${}^{(n+1)}\bar{\sigma}'_{ij} = {}^{(n+1)}\bar{\sigma}'_{ij,\,\text{trial}};\ {}^{(n+1)}\bar{p}_c = {}^{(n)}\bar{p}_c;\ {}^{(n+1)}b = \frac{{}^{(n)}b\,{}^{(n)}\sigma'_{kk}}{{}^{(n+1)}\sigma'_{kk}} \tag{B3}$$

This is the end of the algorithm.

Step-4: plastic corrector. Project the trial stress back onto the bounding surface.

(a) Plastic flow direction (associated flow rule)

$$R_{ij} = L_{ij} = \frac{\partial\bar{F}}{\partial\bar{\sigma}'_{ij}} = 2\ {}^{(n)}s_{ij} + \delta_{ij}\frac{4}{9}M_c^2\left({}^{(n)}b\ {}^{(n)}p' - {}^{(n)}\bar{p}_c\right) \tag{B4}$$

(b) Plastic strain increment

$${}^{(n+1)}\Delta\varepsilon^p_{ij} = \Lambda R_{ij} \tag{B5}$$

(c) Hardening update

$$^{(n+1)}\bar{p}_c = \bar{p}_c \exp\left(\frac{1+e}{\lambda-\kappa}\,^{(n+1)}\Delta\varepsilon_v^p\right) \tag{B6}$$

(d) Actual stress update

$$^{(n+1)}\sigma'_{ij} = {}^{(n)}\sigma'_{ij} + D^e_{ijkl}\left({}^{(n+1)}\Delta\varepsilon_{ij} - {}^{(n+1)}\Delta\varepsilon^p_{ij}\right) \tag{B7}$$

(e) Mapping update

$$^{(n+1)}b = {}^{(n)}b + \frac{{}^{(n)}b^2 K^p_{\mathrm{core}} - {}^{(n)}b\bar{K}^p_{\mathrm{core}}}{{}^{(n)}\bar{p}_c \bar{K}^p_{\mathrm{core}}}\left({}^{(n+1)}\bar{p}_c - {}^{(n)}\bar{p}_c\right) \tag{B8}$$

with plastic modulus K^p_{core} and $\bar{K}^p_{\mathrm{core}}$ being evaluated at the state before update i.e.,

$$\bar{K}^p_{\mathrm{core}} = \bar{K}^p_{\mathrm{core}}\left({}^{(n)}\boldsymbol{\sigma}', {}^{(n)}\bar{p}_c, {}^{(n)}b\right) \tag{B9}$$

$$K^p_{\mathrm{core}} = K^p_{\mathrm{core}}\left(\bar{K}^p_{\mathrm{core}}, {}^{(n)}\bar{p}_c, {}^{(n)}b\right) \tag{B10}$$

(f) Image stress update

$$^{(n+1)}\bar{\sigma}'_{ij} = {}^{(n+1)}b\,{}^{(n+1)}\sigma'_{ij} \tag{B11}$$

(g) Consistent condition on bounding surface

$$\left|\bar{F}\left({}^{(n+1)}\bar{\sigma}'_{ij}, {}^{(n+1)}\bar{p}_c\right)\right| \Big/ {}^{(n+1)}\bar{p}_c \leq \mathrm{FTOL} \tag{B12}$$

where FTOL=1e-6

The system of non-linear equations consists of Eqs. (B4)-(B12) can be solved by the Newton scheme.

This is the end of the algorithm.

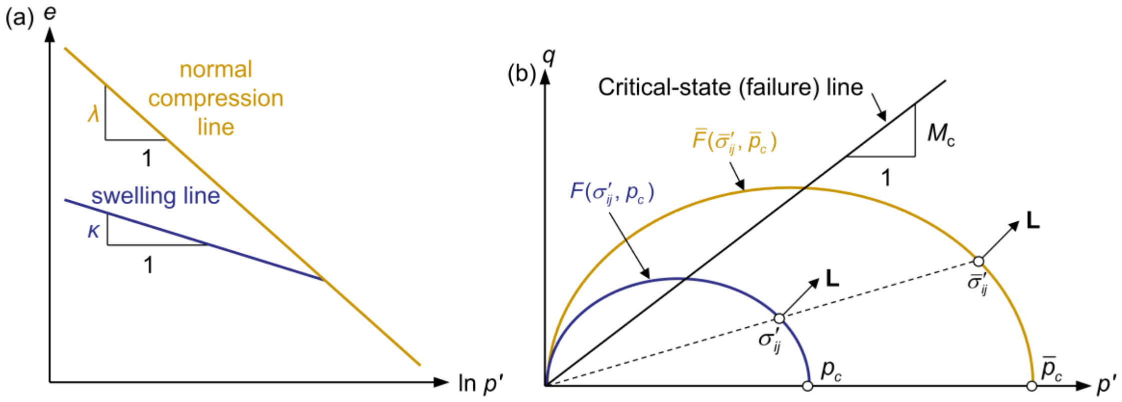

Fig. S1. Interpretation of key constitutive parameters and concepts of the BS model. (**a**) Slopes of normal compression lines (λ) and swelling line (κ) in the void ratio (e) versus logarithm of mean effective stress ($\ln p'$) plane. λ governs plastic modulus, while κ governs elastic modulus. (**b**) Loading surface ($F(\cdot)$), bounding surface ($\bar{F}(\cdot)$), and mapping rule in deviatoric stress (q) versus mean effective stress (p') space. Both $\bar{\sigma}'_{ij} / \sigma'_{ij}$ and p_c are ellipses and geometrically similar about the origin, with the similarity ratio defined by the mapping rule $b = \bar{p}_c$. p_c and $\bar{p}_c$ are the sizes of the loading surface and bounding surface, respectively. L denotes the gradient direction of both surfaces. Mc is the critical state stress ratio (q/p') at failure.

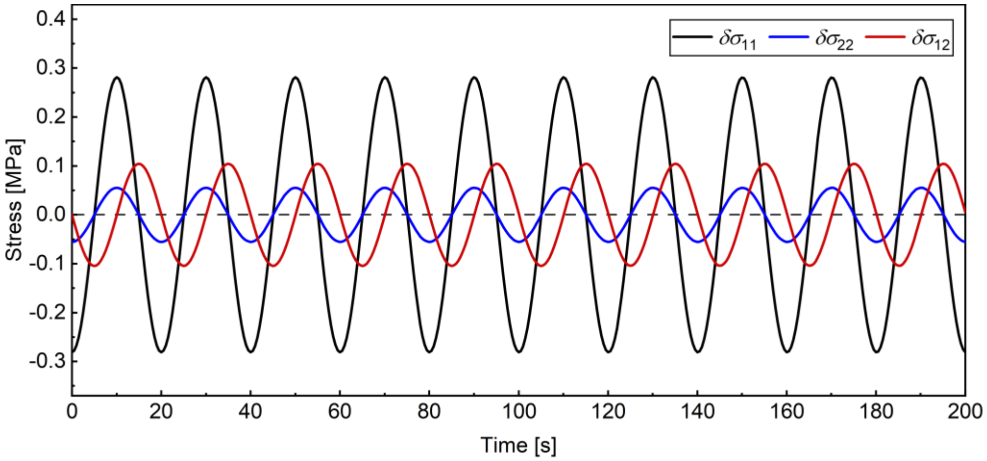

Fig. S2. Time histories of dynamic stress components imposed on fault zone. The black, red, and blue lines donate the horizontal normal stress ($\delta\sigma_{11}$), vertical normal stress ($\delta\sigma_{22}$), and shear tress ($\delta\sigma_{12}$), respectively.

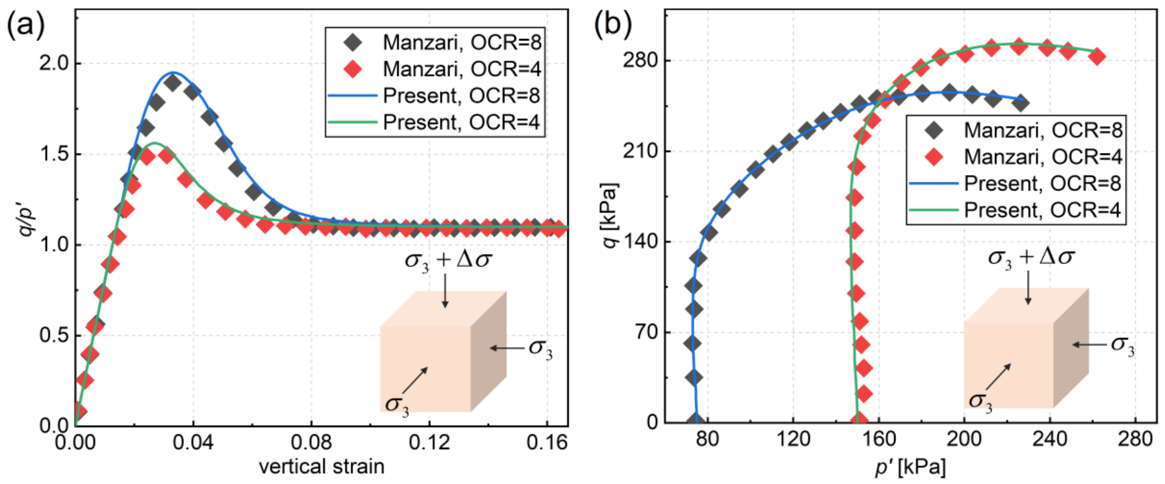

Fig. S3. Comparison of undrained triaxial test results from our numerical framework and from Manzari and Nour (*63*). (**a**) Stress ratio (q/p') versus axial strain. (**b**) Mean normal stress versus deviatoric stress. Rhombus markers indicate results from Manzari and Nour (*63*); solid lines represent our simulation results. The over consolidation ratio (OCR) is defined as the ratio of the maximum historical stress to the current stress. Inset at bottom right shows the test element, where σ_3 and $\Delta\sigma$ denote the confining pressure and deviatoric stress, respectively.

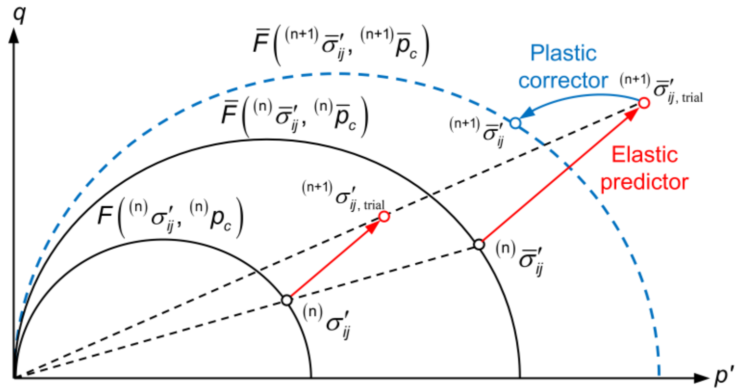

Fig. S4. Schematic of the return mapping algorithm for the BS model. The algorithm comprises an elastic predictor phase followed by a plastic corrector phase. At the start of each load increment, a trial stress (${}^{(n+1)}\sigma'_{ij,\,\mathrm{trial}}$) on the loading surface ($F({}^{(n)}\cdot)$) is calculated by assuming a fully elastic response to the incremental strain. This trial stress is projected onto the bounding surface via the mapping rule, resulting in an image trial stress (${}^{(n+1)}\bar{\sigma}'_{ij,\,\mathrm{trial}}$). If the image trial stress violates the yield criteria ($\bar{F}({}^{(n+1)}\bar{\sigma}'_{ij,\,\mathrm{trial}},\ {}^{(n)}\bar{p}_c) > 0$), a plastic correction step is applied to return the stress state to the bounding surface ($\bar{F}({}^{(n+1)}\cdot)$).

Table S1. Mechanical and physical parameters of fault gouge.

	Symbol	Parameter name	Value
Mechanical parameters	λ	Slope of the normal consolidation line	0.1
	κ	Slope of the swelling line	0.01
	M_c	Stress ratio at critical state	1.4
	υ_{core}	Poisson's ratio	0.3
	H_0	Parameter controlling plastic modulus	20
Physical parameters	ϕ_{core}	Porosity of fault core	0.2
	ρ_{core}	Density of fault core	2500 kg/m^3
	k_{core}	Permeability of fault core	1×10^{-18} m^2
	α_{core}	Biot-Willis's coefficient	1

Table S2. Mechanical and physical parameters of the off-fault zone.

	Symbol	Parameter name	Value
Mechanical parameters	V_P	P wave velocity (drained)	6500 m/s
	V_S	S wave velocity	3800 m/s
Physical parameters	ϕ_{off}	Porosity of off-fault zone	0.1
	ρ_{off}	Density of off-fault zone	2670 kg/m^3
	k_{off}	Permeability of off-fault zone	1×10^{-15} m^2
	α_{off}	Biot-Willis's coefficient	1

Table S3. Parameters of pore fluid.

Symbol	Parameter name	Value
K_f	Fluid bulk modulus	5 GPa
ρ_f	Fluid density	1000 kg/m^3
η_f	Fluid viscosity	0.001 Pa·s

Table S4. Parameters of Rayleigh waves.

Symbol	Parameter name	Value
A_R	Parameter controlling Rayleigh wave amplitude	1500
V_R	Rayleigh wave velocity	3500 m/s
T_R	Rayleigh wave period	20 s

REFERENCES

1. D. P. Hill, P. A. Reasenberg, A. Michael, W. J. Arabaz, G. Beroza, D. Brumbaugh, J. N. Brune, R. Castro, S. Davis, D. dePolo, W. L. Ellsworth, J. Gomberg, S. Harmsen, L. House, S. M. Jackson, M. J. S. Johnston, L. Jones, R. Keller, S. Malone, L. Munguia, S. Nava, J. C. Pechmann, A. Sanford, R. W. Simpson, R. B. Smith, M. Stark, M. Stickney, A. Vidal, S. Walter, V. Wong, J. Zollweg, Seismicity remotely triggered by the magnitude 7.3 Landers, California, earthquake. *Science* **260**, 1617–1623 (1993).

2. A. A. Velasco, S. Hernandez, T. Parsons, K. Pankow, Global ubiquity of dynamic earthquake triggering. *Nat. Geosci.* **1**, 375–379 (2008).

3. C. Aiken, X. F. Meng, J. Hardebeck, Testing for the 'predictability' of dynamically triggered earthquakes in The Geysers geothermal field. *Earth Planet. Sci. Lett.* **486**, 129–140 (2018).

4. V. Sevilgen, R. S. Stein, F. F. Pollitz, Stress imparted by the great 2004 Sumatra earthquake shut down transforms and activated rifts up to 400 km away in the Andaman Sea. *Proc. Natl. Acad. Sci. U.S.A.* **109**, 15152–15156 (2012).

5. Z. Bayramov, R. Viltres, C. Doubre, A. Maggi, R. Jolivet, L. Rivera, Very-long-range dynamic triggering of mud volcano unrest and silent magnitude-6 fault slip. *Science* **389**, 996–1002 (2025).

6. E. E. Brodsky, N. J. van der Elst, The uses of dynamic earthquake triggering. *Annu. Rev. Earth Planet. Sci.* **42**, 317–339 (2014).

7. T. Parsons, A. A. Velasco, Absence of remotely triggered large earthquakes beyond the mainshock region. *Nat. Geosci.* **4**, 312–316 (2011).

8. E. E. Brodsky, The importance of studying small earthquakes. *Science* **364**, 736–737 (2019).

9. J. G. Anderson, J. N. Brune, J. N. Louie, Y. Zeng, M. Savage, G. Yu, Q. Chen, D. dePolo, Seismicity in the western Great Basin apparently triggered by the Landers, California, earthquake, 28 June 1992. *Bull. Seismol. Soc. Am.* **84**, 863–891 (1994).

10. K. R. Felzer, E. E. Brodsky, Decay of aftershock density with distance indicates triggering by dynamic stress. *Nature* **441**, 735–738 (2006).

11. J. Gomberg, P. Johnson, Seismology—Dynamic triggering of earthquakes. *Nature* **437**, 830–830 (2005).

12. W. Fan, P. M. Shearer, Local near instantaneously dynamically triggered aftershocks of large earthquakes. *Science* **353**, 1133–1136 (2016).

13. A. F. Bell, S. Hernandez, J. McCloskey, M. Ruiz, P. C. LaFemina, C. J. Bean, M. Möllhoff, Dynamic earthquake triggering response tracks evolving unrest at Sierra Negra volcano, Galápagos Islands. *Sci. Adv.* **7**, eabh0894 (2021).

14. A. M. Freed, Earthquake triggering by static, dynamic, and postseismic stress transfer. *Annu. Rev. Earth Planet. Sci.* **33**, 335–367 (2005).

15. A. R. Bansal, A. Ghods, Remote triggering in Iran: Large peak dynamic stress is not the main driver of triggering. *Geophys. J. Int.* **225**, 456–476 (2021).

16. C. H. Scholz, Earthquakes and friction laws. *Nature* **391**, 37–42 (1998).

17. N. Beeler, D. Lockner, Why earthquakes correlate weakly with the solid Earth tides: Effects of periodic stress on the rate and probability of earthquake occurrence. *J. Geophys. Res. Solid Earth* **108**, 2391 (2003).

18. E. E. Brodsky, S. G. Prejean, New constraints on mechanisms of remotely triggered seismicity at Long Valley Caldera. *J. Geophys. Res.* **110**, B04302 (2005).

19. H. Lestrelin, J.-P. Ampuero, E. D. Mercerat, F. Courboulex, Modeling the onset of earthquake-triggered landslides on slip surfaces governed by rate-and-state friction. *Geophys. Res. Lett.* **51**, e2024GL110695 (2024).

20. D. P. Hill, Dynamic stresses, Coulomb failure, and remote triggering. *Bull. Seismol. Soc. Am.* **98**, 66–92 (2008).

21. M. D. Zoback, M. L. Zoback, Stress in the Earth's lithosphere. *Encycl. Phys. Sci. Technol.* **16**, 143–154 (2002).

22. D. P. Hill, S. Prejean, Dynamic triggering. *Treatise Geophys.* **4**, 257–291 (2007).

23. F. F. Pollitz, R. S. Stein, V. Sevilgen, R. Bürgmann, The 11 April 2012 east Indian Ocean earthquake triggered large aftershocks worldwide. *Nature* **490**, 250–253 (2012).

24. S. Ruiz, M. Metois, A. Fuenzalida, J. Ruiz, F. Leyton, R. Grandin, C. Vigny, R. Madariaga, J. Campos, Intense foreshocks and a slow slip event preceded the 2014 Iquique M_w 8.1 earthquake. *Science* **345**, 1165–1169 (2014).

25. T. Parsons, A hypothesis for delayed dynamic earthquake triggering. *Geophys. Res. Lett.* **32**, L04302 (2005).

26. A. L. Handwerger, A. W. Rempel, R. M. Skarbek, J. J. Roering, G. E. Hilley, Rate-weakening friction characterizes both slow sliding and catastrophic failure of landslides. *Proc. Natl. Acad. Sci. U.S.A.* **113**, 10281–10286 (2016).

27. T. Parsons, L. Malagnini, A. Akinci, Nucleation speed limit on remote fluid-induced earthquakes. *Sci. Adv.* **3**, e1700660 (2017).

28. N. J. Van der Elst, H. M. Savage, K. M. Keranen, G. A. Abers, Enhanced remote earthquake triggering at fluid-injection sites in the midwestern United States. *Science* **341**, 164–167 (2013).

29. R. Alfaro-Diaz, A. A. Velasco, K. L. Pankow, D. Kilb, Optimally oriented remote triggering in the Coso Geothermal Region. *J. Geophys. Res.: Solid Earth* **125**, e2019JB019131 (2020).

30. A. Guilhem, Z. G. Peng, R. M. Nadeau, High-frequency identification of non-volcanic tremor triggered by regional earthquakes. *Geophys. Res. Lett.* **37**, L16309 (2010).

31. D. R. Shelly, Z. G. Peng, D. P. Hill, C. Aiken, Triggered creep as a possible mechanism for delayed dynamic triggering of tremor and earthquakes. *Nat. Geosci.* **4**, 384–388 (2011).

32. G. Farge, E. E. Brodsky, The big impact of small quakes on tectonic tremor synchronization. *Sci. Adv.* **11**, eadu7173 (2025).

33. A. H. Lachenbruch, Frictional heating, fluid pressure, and the resistance to fault motion. *J. Geophys. Res.: Solid Earth* **85**, 6097–6112 (1980).

34. T. Ishikawa, T. Hirono, N. Matsuta, K. Kawamoto, K. Fujimoto, J. Kameda, Y. Nishio, Y. Maekawa, G. Honda, Geochemical and mineralogical characteristics of fault gouge in the Median Tectonic Line, Japan: Evidence for earthquake slip. *Earth Planets Space* **66**, 36 (2014).

35. C. Marone, C. H. Scholz, Particle-size distribution and microstructures within simulated fault gouge. *J. Struct. Geol.* **11**, 799–814 (1989).

36. M. J. Ikari, D. M. Saffer, C. Marone, Frictional and hydrologic properties of clay-rich fault gouge. *J. Geophys. Res.: Solid Earth* **114**, B05409 (2009).

37. D. R. Faulkner, C. Sanchez-Roa, C. Boulton, S. Den Hartog, Pore fluid pressure development in compacting fault gouge in theory, experiments, and nature. *J. Geophys. Res. Solid Earth* **123**, 226–241 (2018).

38. F. Chester, J. M. Logan, Implications for mechanical properties of brittle faults from observations of the Punchbowl fault zone, California. *Pure Appl. Geophys.* **124**, 79–106 (1986).

39. C. K. Lieou, A. E. Elbanna, J. Langer, J. Carlson, Shear flow of angular grains: Acoustic effects and nonmonotonic rate dependence of volume. *Phys. Rev. E* **90**, 032204 (2014).

40. K. Farain, D. Bonn, Perturbation-induced granular fluidization as a model for remote earthquake triggering. *Sci. Adv.* **10**, eadi7302 (2024).

41. L. Callisto, G. Calabresi, Mechanical behaviour of a natural soft clay. *Géotechnique* **48**, 495–513 (1998).

42. J. Yang, H. Sze, Cyclic behaviour and resistance of saturated sand under non-symmetrical loading conditions. *Géotechnique* **61**, 59–73 (2011).

43. J. Yang, T. Sato, Interpretation of seismic vertical amplification observed at an array site. *Bull. Seismol. Soc. Am.* **90**, 275–285 (2000).

44. D. P. Hill, On the sensitivity of transtensional versus transpressional tectonic regimes to remote dynamic triggering by Coulomb failure. *Bull. Seismol. Soc. Am.* **105**, 1339–1348 (2015).

45. D. P. Hill, S. Prejean, Dynamic triggering. *Treatise Geophys.* **4**, 273–304 (2015).

46. B. Enescu, K. Shimojo, A. Opris, Y. Yagi, Remote triggering of seismicity at Japanese volcanoes following the 2016 M7. 3 Kumamoto earthquake. *Earth Planets Space* **68**, 165 (2016).

47. M. G. Miller, Ductility in fault gouge from a normal fault system, Death Valley, California: A mechanism for fault-zone strengthening and relevance to paleoseismicity. *Geology* **24**, 603–606 (1996).

48. K. H. Roscoe, A. N. Schofield, C. P. Wroth, On the yielding of soils. *Géotechnique* **8**, 22–53 (1958).

49. E. Hirakawa, S. Ma, Dynamic fault weakening and strengthening by gouge compaction and dilatancy in a fluid-saturated fault zone. *J. Geophys. Res. Solid Earth* **121**, 5988–6008 (2016).

50. Y. F. Dafalias, Bounding surface plasticity. I: Mathematical foundation and hypoplasticity. *J. Eng. Mech.* **112**, 966–987 (1986).

51. S. Prejean, D. Hill, E. Brodsky, S. Hough, M. Johnston, S. Malone, D. Oppenheimer, A. Pitt, K. Richards-Dinger, Remotely triggered seismicity on the United States west coast following the M_w 7.9 Denali fault earthquake. *Bull. Seismol. Soc. Am.* **94**, S348–S359 (2004).

52. M. D. Zoback, 34 - State of stress in the Earth’s lithosphere. *Int. Geophys.* **81**, 559–568 (2002).

53. N. D. DeSalvio, W. Fan, Ubiquitous earthquake dynamic triggering in southern California. *J. Geophys. Res. Solid Earth* **128**, e2023JB026487 (2023).

54. C. A. Morrow, J. D. Byerlee, Experimental studies of compaction and dilatancy during frictional sliding on faults containing gouge. *J. Struct. Geol.* **11**, 815–825 (1989).

55. M. Hattab, P.-Y. Hicher, Dilating behaviour of overconsolidated clay. *Soils Found.* **44**, 27–40 (2004).

56. J. Yang, X. Li, State-dependent strength of sands from the perspective of unified modeling. *J. Geotech. Geoenviron. Eng.* **130**, 186–198 (2004).

57. J. J. Sánchez, S. R. McNutt, Intermediate-term declines in seismicity at Mt. Wrangell and Mt. Veniaminof volcanoes, Alaska, following the 3 November 2002 M_w 7.9 Denali fault earthquake. *Bull. Seismol. Soc. Am.* **94**, S370–S383 (2004).

58. P. G. Okubo, C. J. Wolfe, Swarms of similar long-period earthquakes in the mantle beneath Mauna Loa Volcano. *J. Volcanol. Geotherm. Res.* **178**, 787–794 (2008).

59. L. Han, Z. Peng, C. W. Johnson, F. F. Pollitz, L. Li, B. Wang, J. Wu, Q. Li, H. Wei, Shallow microearthquakes near Chongqing, China triggered by the Rayleigh waves of the 2015 M7. 8 Gorkha, Nepal earthquake. *Earth Planet. Sci. Lett.* **479**, 231–240 (2017).

60. R. Alfaro-Diaz, A. A. Velasco, D. L. Guenaga, Insights from dynamically triggered and induced earthquakes in Oklahoma. *Seismol. Soc. Am.* **94**, 685–698 (2023).

61. C. J. Ammon, A. A. Velasco, T. Lay, T. C. Wallace, *Foundations of Modern Global Seismology* (Academic Press, 2021).

62. M. Lupi, M. Frehner, P. Weis, A. Skelton, E. H. Saenger, N. Tisato, S. Geiger, G. Chiodini, T. Driesner, Regional earthquakes followed by delayed ground uplifts at Campi Flegrei Caldera, Italy: Arguments for a causal link. *Earth Planet. Sci. Lett.* **474**, 436–446 (2017).

63. M. Manzari, M. Nour, On implicit integration of bounding surface plasticity models. *Comput. Struct.* **63**, 385–395 (1997).

64. L. Cueto-Felgueroso, D. Santillán, J. C. Mosquera, Stick-slip dynamics of flow-induced seismicity on rate and state faults. *Geophys. Res. Lett.* **44**, 4098 – 4106 (2017).

65. B. Crawford, D. Faulkner, E. Rutter, Strength, porosity, and permeability development during hydrostatic and shear loading of synthetic quartz-clay fault gouge. *J. Geophys. Res. Solid Earth* **113**, B03207 (2008).
